# Bilobular calcifying fibrous pseudotumor in soleus muscle: a case report

**DOI:** 10.1186/1752-1947-5-487

**Published:** 2011-09-28

**Authors:** Naohiro Shinohara, Satoshi Nagano, Masahiro Yokouchi, Yoshiya Arishima, Kazuhiro Tabata, Michiyo Higashi, Shinichi Kitajima, Suguru Yonezawa, Setsuro Komiya

**Affiliations:** 1 Department of Orthopaedic Surgery, Graduate School of Medical and Dental Sciences, Kagoshima University, Kagoshima, Japan; 2 Department of Human Pathology, Graduate School of Medical and Dental Sciences, Kagoshima University, Kagoshima, Japan; 3 Division of Surgical Pathology, Kagoshima University Hospital, Kagoshima, Japan

## Abstract

**Introduction:**

Calcifying fibrous pseudotumor is a rare benign soft-tissue lesion composed of fibrous tissue with abundant hyalinized collagen and dystrophic and often psammomatous calcifications. The cause of the disease is unclear but, usually, complete resection of the well-circumscribed tumor is sufficient to avoid recurrence of the disease. Here, we report an unusual case of this rare tumor that presented as two lobulated lesions in the calf muscle.

**Case presentation:**

The patient was a 17-year-old Japanese girl who noted a hard mass in her left calf. Magnetic resonance imaging revealed two well-demarcated lobular masses in the soleus muscle, and the tumor was significantly enhanced by contrast medium. Preoperative differential diagnoses included soft-part tumors composed of fibrous tissue. However, making a definite diagnosis was impossible because a lobulated shape is rare for fibrous tumors. Biopsy demonstrated that the mass was a benign tumor composed of collagen-rich, hyalinized fibrosclerotic tissue. We performed marginal resection of the two nodules, including the fibrous tissue that connected them. Immunohistochemistry was positive for factor XIIIa and negative for anaplastic lymphoma kinase-1. These findings were helpful to distinguish calcifying fibrous pseudotumor from inflammatory myofibroblastic tumor. There was no sign of recurrence at 30 months after surgery.

**Conclusion:**

To the best of our knowledge, this is the first case of bilobular calcifying fibrous pseudotumor that developed in an extremity. As described in the previous literature, simple excision was sufficient for the treatment of calcifying fibrous pseudotumor with two lobules.

## Introduction

Calcifying fibrous pseudotumor (CFPT) is a rare, benign lesion that was first reported by Rosenthal and Abdul-Karim as "childhood fibrous tumor with psammoma bodies" [1]. Fetsch *et al. *summarized 10 cases and designated the tumor as CFPT in 1993 [2]. Calcifying fibrous pseudotumor mainly occurs in deep soft tissues of children and adolescents[3,4]. Histologically, CFPT is composed of fibrous tissue with diffuse calcification and infiltrating inflammatory cells. Owing to the rarity of the disease, differential diagnosis of CFPT includes a list of diseases [5]. Here, we report the case of a girl with two neighboring lesions in her calf, which were diagnosed as CFPT by biopsy. Immunohistochemistry was performed to differentiate between histologically similar diseases. To the best of our knowledge, this is the first case of bilobular CFPT that developed in an extremity.

## Case presentation

A 17-year-old Japanese girl noted a painless mass in her left calf. Seven months later, because the mass gradually increased in size, she visited our department. Our patient had no history of major traumatic injury. There was no family history or relevant medical history. Physical examination revealed a 3 cm × 3 cm hard, mobile, tender mass in her soleus muscle. No Tinel-like sign was elicited. Blood examination, including inflammatory response, was negative.

**Figure 1:**
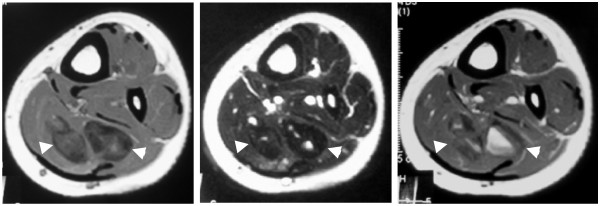
**Magnetic resonance images of the lesion**. T1-weighted images showed a mixture of areas of isointense and low signal intensity (left panel). T2-weighted images showed areas of low signal intensity (middle panel). On gadolinium-enhanced T1-weighted images, the lateral mass showed significant enhancement (right panel).

Plain radiographs did not reveal any abnormal shadow. Magnetic resonance imaging (MRI) revealed two well-demarcated lobular masses in her soleus muscle (Figure 1). T1-weighted images showed a mixture of areas of signal intensity equal to that of the surrounding muscles and areas of low signal intensity. T2-weighted images showed areas of low signal intensity, suggestive of a tumor containing fibrous tissue. On gadolinium-enhanced T1-weighted images, the lateral mass showed significant enhancement, whereas the medial mass presented irregular focal areas of enhancement. Preoperative differential diagnoses included solitary fibrous tumor and inflammatory myofibroblastic tumor (IMT). Since MRI showed contrast enhancement, a malignant tumor such as fibrosarcoma or synovial sarcoma could not be completely excluded. Two months after the initial visit to our department, an open biopsy was performed, and a sample of yellowish-white, solid, fibrous tissue was obtained. Microscopically, the sample was composed of collagen-rich, hyalinized fibrosclerotic tissue and infiltrated inflammatory cells. Calcifying fibrous pseudotumor was the most likely pathological diagnosis. There was no evidence of malignancy.

**Figure 2:**
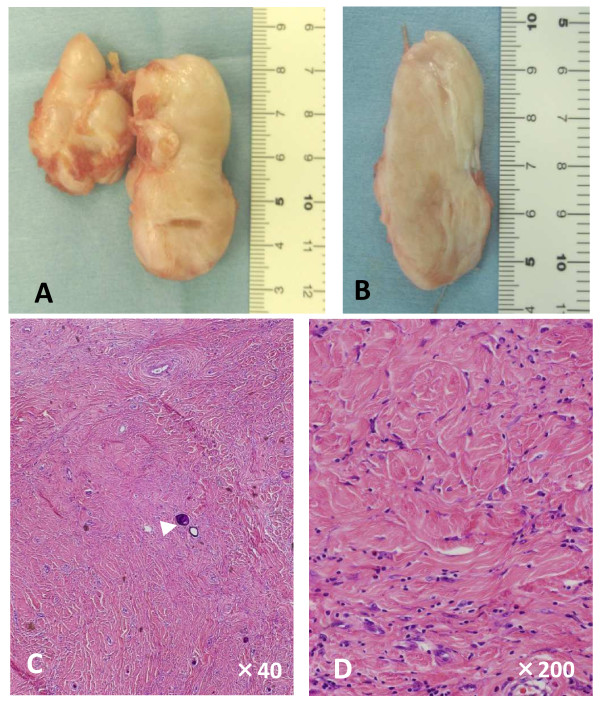
**Macroscopic (A and B) and microscopic (C and D) features of the resected mass**. The two lesions were found to be solid, lobular, well-circumscribed, and gray-white masses. **(A) **The two nodules were connected with each other by fibrous tissue. **(B) **The cut surface was gray-white and homogenous. **(C, D) **Hematoxylin and eosin staining of the lesions revealed that they were composed of hyalinized collagen fibers with fibroblasts. Psammomatous calcifications were observed (C, arrowhead). Original magnifications: (C) ×40; (D) ×200.

Because the tumor was considered benign, simple excision of the mass was performed two months after the biopsy. At the time of excision, two masses were found in her soleus muscle. There was no feeding vessel and no hemorrhage originated from the tumor. On gross examination, the lesions were found to be solid, lobular, well-circumscribed, non-encapsulated, and gray-white masses (Figure 2A). The two lesions were connected by fibrous tissue. The cut surface was gray-white and homogenous (Figure 2B).

Microscopically, the lesion was composed of hyalinized collagen fibers with fibroblasts. Psammomatous calcifications and infiltration of inflammatory cells were observed (Figure 2C and 2D). Immunostaining was positive for factor XIIIa and CD34, rarely positive for Ki-67, and negative for anaplastic lymphoma kinase (ALK).

Our patient was discharged without having experienced any postoperative complications. She could walk without any pain or functional disability. At 30 months after surgery, there was no evidence of recurrence.

## Discussion

Calcifying fibrous pseudotumor is a rare, benign, tumor-like lesion characterized histologically by fibrotic proliferation, infiltration of inflammatory cells, and dystrophic and/or psammomatous calcifications [2,5]. The lesion has been found in a wide range of anatomic sites, including the extremities, trunk, neck, mesenterium, mediastinum and paratesticular area [2,4]. Previous case reports showed that multiple CFPT may also develop in the pleura or abdominal cavity [6-9]. To the best of our knowledge, this is the first case of bilobular CFPT that developed in an extremity. Because of the rarity of CFPT and unusual presentation of the present case, we did not consider CFPT as the most suspected diagnosis. Radiologic differential diagnosis of CFPT includes fibromatosis, IMT, fibrosarcoma or low-grade fibromyxoid sarcoma. Our case showed two neighboring fibrous nodules on MRI, which made a definitive preoperative diagnosis difficult. The biopsy specimen revealed no evidence of malignancy and CFPT was the most likely diagnosis. Although there was no literature reporting the surgical treatment of bilobular CFPT, we performed marginal resection, including the connecting tissues. The main differential diagnosis to CFPT is IMT, which has a tendency of local recurrence [5,10]. A review of the literature also suggested that simple excision with a margin of normal tissue is sufficient for CFPT [5], whereas some reports suggested wide margins for IMT. Recurrence of CFPT has been noted in only four patients; however, the authors speculated that incomplete excision had occurred in those cases [11]. Our case presented two nodules connected with each other, which may reflect an inflammatory origin of the tumor. In spite of the unusual presentation of the tumor, there was no evidence of recurrence at two and a half years after surgery. Therefore, we consider that complete resection, including the connective tissue between the nodules, is sufficient for the treatment of bilobular CFPT.

Pathological differentiation of CFPT from IMT is sometimes problematic because spindle cells, inflammatory cell infiltration and calcification are common in both entities. Some literature has proposed that CFPT is a sclerosing end-stage of IMT [10]. Immunohistological staining is helpful to distinguish the two diseases. Sigel *et al. *noted that ALK positivity is often observed in IMT but absent in CFPT [12]. Chromosomal translocations have been reported to lead to the activation of ALK in IMTs, particularly in those arising in young patients [13]. Recently, Agaimy *et al. *reported a molecular study of seven cases of gastric CFPT and addressed the issue of immunoglobulin G4 and the lacking recurrence in contrast to soft tissue counterparts [14]. Factor XIIIa, a protransglutaminase synthesized in the liver, functions in the coagulation pathway via stabilization of clot formation. Hill *et al. *showed diffuse staining for factor XIIIa in CFPT, in contrast to focal staining in IMT [15]. They also described strong reactivity of CFPT to factor XIIIa and suggested that this tumor may be considered "fibrohistiocytic" in origin. In our case, staining for factor XIIIa was diffusely positive, suggestive of CFPT rather than IMT.

## Conclusion

In conclusion, we report the first case of bilobular CFPT in an extremity. Immunostaining for ALK and factor XIIIa was useful to differentiate between CFPT and IMT. Bilobular CFPT can be successfully treated by marginal resection.

## Consent

Written informed consent was obtained from the patient's parents for publication of this case report and any accompanying images. A copy of the written consent is available for review by the Editor-in-Chief of this journal.

## Competing interests

The authors declare that they have no competing interests.

## Authors' contributions

NS and SN performed the assessment and treatment of the patient and wrote the manuscript. MY and YA participated in the treatment of the patient, including surgery. KT, MH, SKi, and SY carried out the histological study. SKo designed the study and drafted the manuscript. All authors read and approved the final manuscript.
